# The gut microbiota in osteoporosis: dual roles and therapeutic prospects

**DOI:** 10.3389/fimmu.2025.1617459

**Published:** 2025-09-01

**Authors:** Xingwen Xie, Hao Liu, Kangwei Wan, Jiawen Li, Peng Qi

**Affiliations:** ^1^ Affiliated Hospital of Gansu University of Traditional Chinese Medicine, Lanzhou, China; ^2^ Gansu University of Traditional Chinese Medicine, Lanzhou, China

**Keywords:** gut-bone axis, short-chain fatty acids, osteoimmunology, FMT, probiotics, bone mineral density

## Abstract

Recent advances in bone biology have underscored the essential role of the gut microbiota in maintaining skeletal homeostasis. Gut-derived metabolites, particularly short chain fatty acids and tryptophan derivatives, influence bone metabolism through modulation of immune signaling, inflammation, and endocrine networks. Emerging evidence indicates that these effects are context dependent and dose dependent, rather than uniformly beneficial or detrimental. For instance, butyrate and lipopolysaccharide exhibit biphasic effects on both osteogenesis and osteoclastogenesis, contingent on concentration, immune status, and the local microenvironment. Microbiota-targeted strategies such as probiotics, prebiotics, and fecal microbiota transplantation are under active investigation as innovative interventions for osteoporosis in both preclinical and clinical contexts. However, substantial knowledge gaps persist, including inconsistent therapeutic outcomes, limited mechanistic insight into host–microbiota interactions, and the absence of standardized microbial intervention protocols. In addition, safety concerns related to FMT, particularly in immunocompromised elderly populations, emphasize the need for rigorous donor screening, extended follow-up periods, and personalized risk and benefit assessment models. To advance the field, future studies should incorporate multi-omics platforms and precision medicine tools to identify key microbial targets and enhance therapeutic efficacy. This review consolidates current evidence and proposes a conceptual framework to clarify the context-specific roles of the gut microbiota in bone remodeling. A deeper mechanistic understanding will be crucial for translating microbiota-based strategies into safe and effective treatments for metabolic bone disorders.

## 1 Introduction

Osteoporosis constitutes a prevalent metabolic bone disorder characterized by diminished bone mass, compromised microarchitecture, and elevated fracture susceptibility (1). With an annual global incidence exceeding 100 million cases and a male-to-female prevalence ratio of approximately 2:3, this condition represents a growing healthcare challenge. Population aging trends compound the osteoporotic burden, compromising patient quality of life while imposing substantial healthcare costs (2, pp. 2005–2010). The insidious nature of early-stage disease, presenting with subtle pain and spinal deformities, often delays diagnosis and increases fracture risk (3).

Human microbiota complexity encompasses diverse microbial ecosystems, with the gastrointestinal tract harboring approximately 70% of total microbial biomass (4, 5). The gut microbiota represents the body’s most extensive microbial ecosystem, containing up to 10^14 microorganisms whose collective genome exceeds human genetic content by 150-fold (6–8). This “microbial organ” maintains critical physiological functions including intestinal barrier integrity, nutrient metabolism, and immunometabolic homeostasis (9, 10). The human gut microbiota comprises predominantly *Firmicutes, Bacteroidetes, Actinobacteria*, and *Proteobacteria* phyla, orchestrating essential processes encompassing nutrient biotransformation, xenobiotic detoxification, immune modulation, and barrier function maintenance (11, 12). Accumulating evidence establishes sophisticated bidirectional communication networks between gut microbiota and skeletal systems, termed the “gut-bone axis” (13). Microbial communities and their metabolic derivatives regulate bone homeostasis through direct and indirect mechanisms, modulating osteoblast-osteoclast dynamics via metabolic, inflammatory, and immune pathways (14). Physiological microbiota stability promotes immune equilibrium, supporting osteocyte function and balanced bone turnover (15, 16). Conversely, microbial dysbiosis disrupts skeletal homeostasis through multiple mechanisms: immune system perturbation altering *CD4+* T cell subset ratios and cytokine profiles, particularly *receptor activator of nuclear factor kappa-B ligand (RANKL)* expression, thereby promoting osteoclastogenesis (15); direct metabolite-mediated regulation of osteoblast and osteoclast differentiation through *short chain fatty acids (SCFAs)* and bile acid signaling (17); and dysbiosis-induced inflammatory cascades releasing bone-catabolic mediators that disrupt formation-resorption coupling (18). Reciprocally, bone-derived hormones and extracellular vesicles modulate intestinal epithelial function, influencing barrier integrity and microbial homeostasis. Skeletal metabolic perturbations can trigger intestinal stress responses, promoting epithelial damage and microbiota dysbiosis, which perpetuates inflammatory bone loss (19). Elucidating these complex microbiota-bone interactions therefore holds profound therapeutic implications for developing targeted interventions against metabolic bone diseases.

## 2 Gut microbiota and metabolite associations with osteoporosis

**Figure 1:**
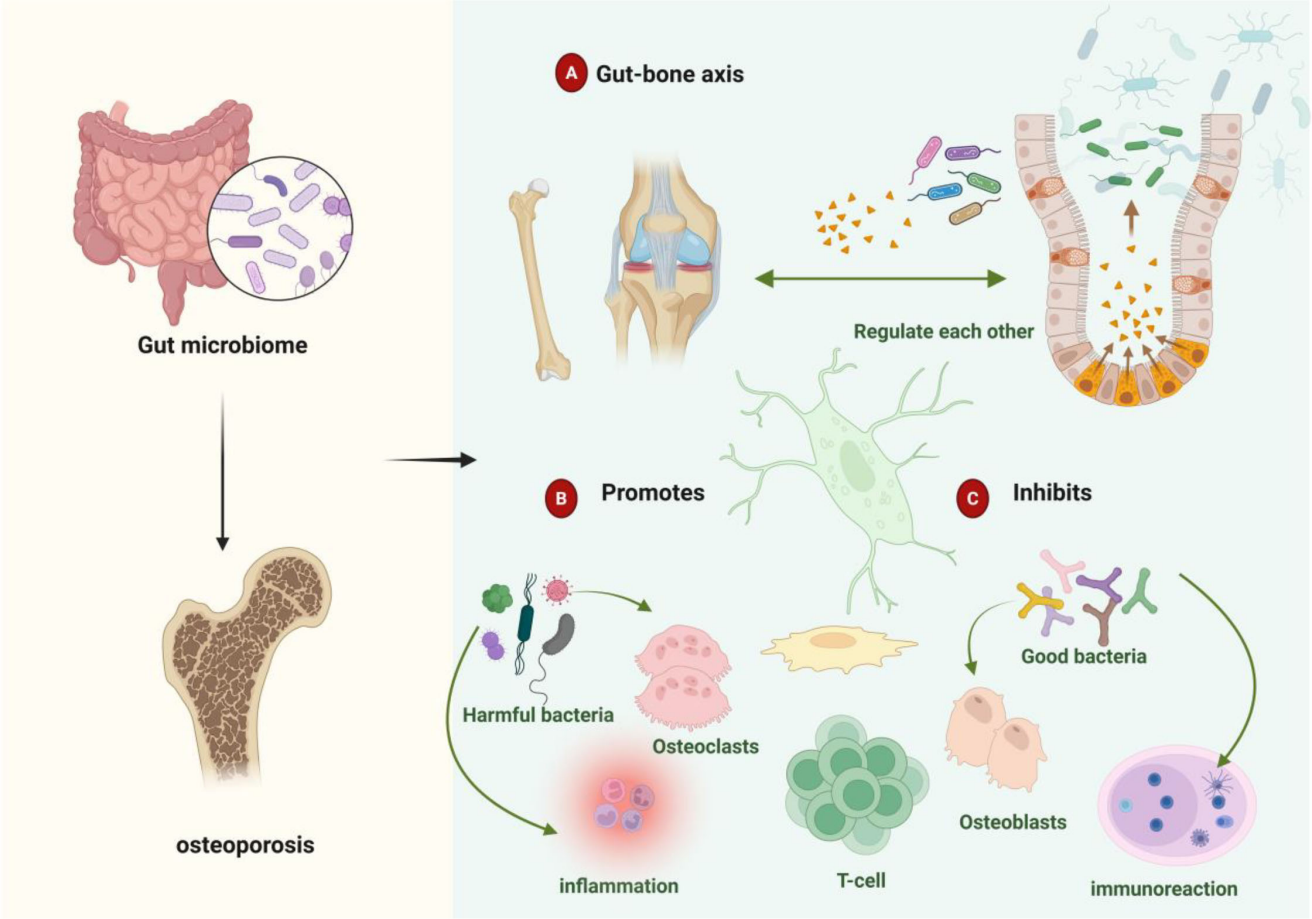
Gut microbiota-metabolite associations with osteoporosis. **(A)** Bidirectional gut-bone axis communication mediates skeletal homeostasis through direct and indirect microbiota-derived signals. **(B)** Dysbiotic conditions promote pathogenic bacterial expansion, accelerating osteoclastogenesis and inflammatory bone loss. **(C)** Beneficial microbes and their metabolites maintain skeletal integrity through immunomodulatory mechanisms that favor bone formation over resorption.

Patients with osteoporosis exhibit pronounced gut microbiota dysbiosis characterized by markedly reduced microbial community diversity (20). **Figure 1** illustrates the mechanistic associations between gut microbiota, their metabolites, and osteoporosis pathogenesis. microbiota analyses demonstrate elevated abundances of *Bacteroidetes, Bacteroides* and *Eisenbergiella*, alongside increased abundances of *Clostridium, Lactobacillus*, and *Eggerthella* species in osteoporotic individuals. Concurrently, relative abundances of Parabacteroides and *Flavobacterium* increase while Ruminococcus decreases significantly (21). Chen et al. (22) identified substantial reductions in lactobacilli and butyrate-producing bacterial populations. Paradoxically, they observed elevated concentrations of osteogenic metabolites—alkaline phosphatase, runt-related transcription factor 2, and osteoprotegerin—derived from *Lactobacillus acidophilus*, L. rhamnosus, and butyrate-producing species. These metabolites collectively enhance osteoblast proliferation and differentiation.

Bacterial taxa demonstrate distinct regulatory effects on bone homeostasis. Beneficial commensals, including *Bifidobacterium* sp*ecies*, promote regulatory T cells (Treg) differentiation while suppressing excessive bone resorption (23). Conversely, pathogenic bacteria—notably segmented filamentous bacteria and certain Ruminococcus species—exacerbate osteoclastogenesis and bone catabolism through T helper 17 cells (Th17) activation (24, 25). Microbial metabolites serve as critical mediators: butyrate stimulates osteoblast differentiation and bone accrual, whereas *lipopolysaccharide (LPS)* inhibits osteoblast maturation while promoting osteoclast activation, collectively influencing osteoporotic progression. Investigation of gut virome and mycobiome effects on skeletal health remains in its infancy. These microbial communities modulate both intestinal barrier integrity and bone metabolism through sophisticated immunoregulatory networks (26). Fungal species, particularly *Candida* and *Aspergillus*, indirectly influence bone turnover by modulating host immune responses and osteoclast activity (27). However, the precise mechanisms underlying mycobiome-bone interactions require further elucidation. Epidemiological evidence establishes robust associations between gut microbiota dysbiosis and accelerated bone loss, leading to elevated osteoporotic fracture risk (28, 29). Microbial metabolites predominantly derive from incompletely digested dietary substrates and host-secreted mucins (30, 31). These include *SCFAs*, bile acids, indole derivatives, lipopolysaccharide, vitamins, and polyamines (32, 33). Such bioactive compounds orchestrate complex regulatory networks affecting endocrine signaling, immune cell differentiation, inflammatory cascades, and oxidative stress responses, collectively modulating osteoporotic pathogenesis (34). Host factors—including health status, age, sex, and immunological competence—critically determine microbiota-mediated skeletal effects. Age-related immune senescence and microbiota compositional shifts may fundamentally alter metabolite bioactivity, thereby disrupting bone homeostasis. In elderly populations, dysbiotic conditions can transform typically osteoprotective metabolites into bone-catabolic mediators, accelerating resorptive processes and osteoporotic progression.

## 3 Mechanistic pathways of gut microbiota-mediated osteoporotic progression

**Figure 2:**
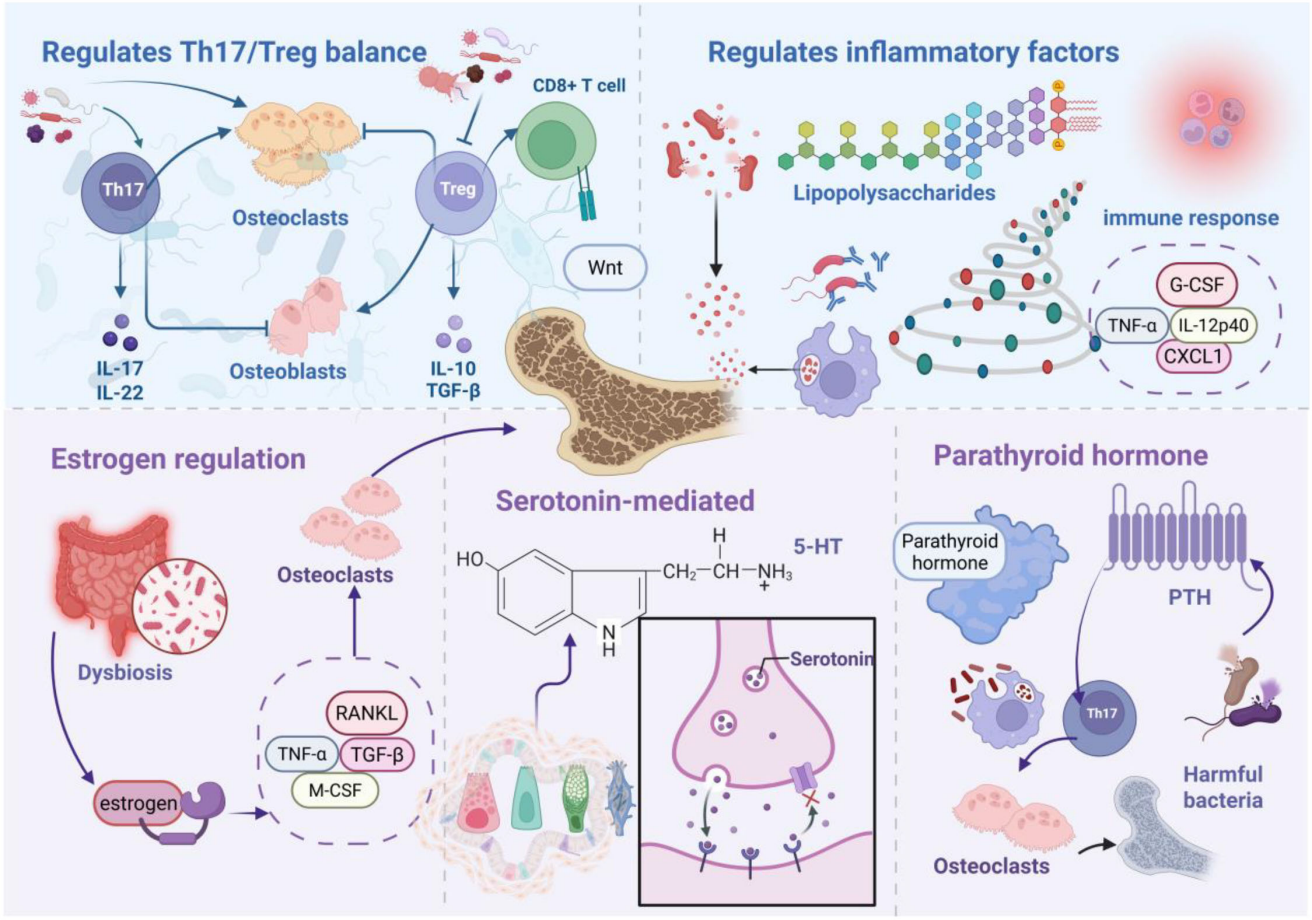
Gut microbiota-driven osteoporotic mechanisms. Microbial dysbiosis promotes pathogenic *bacterial* expansion, with resulting microorganisms and metabolites orchestrating osteoporotic progression through immune cell modulation, Th17/Treg imbalance, and inflammatory mediator dysregulation. Additionally, microbiota-derived signals drive skeletal catabolism via endocrine pathway perturbations, including estrogen, serotonin, and parathyroid hormone (PTH) dysregulation.

Gut microbiota orchestrate complex physiological networks encompassing metabolic regulation, biosynthetic processes, and immunomodulation (35), fundamentally influencing disease pathogenesis across multiple organ systems. In osteoporotic pathophysiology, gut microbial communities and their bioactive metabolites drive skeletal deterioration through sophisticated regulatory mechanisms targeting bone metabolism, immune cell dynamics, and endocrine signaling networks. **Figure 2** illustrates the mechanistic framework whereby gut microbiota promote osteoporotic progression.

### 3.1 Immunoregulatory mechanisms

Gut microbial communities modulate bone tissue immune architecture through sophisticated interactions with intestinal dendritic cells and immune effectors, thereby controlling skeletal homeostasis (36, 37). Germ-free mouse studies demonstrate profound reductions in osteoclast populations alongside diminished bone expression of pro-inflammatory mediators Interleukin-6, *tumor necrosis factor alpha (TNF-α)*, and *RANKL.* microbiota reconstitution restores immune functionality, establishing gut microbiota as fundamental regulators of bone-immune crosstalk (38).

#### 3.1.1 Th17/Treg axis modulation

Th17 cells drive osteoclastogenesis via *RANKL*-dependent pathways (39). Bone marrow-resident Th17 *TNF-α+* populations promote osteoclast differentiation independently of exogenous osteoclastogenic stimuli while stimulating mesenchymal stem cells to secrete chemokines monocyte chemoattractant protein-1, macrophage Inflammatory protein-1 alpha, and *RANKL*, facilitating inflammatory monocyte recruitment and bone catabolism. Zaiss and colleagues elucidated Treg functions in skeletal homeostasis, particularly through osteoclastogenesis suppression. This seminal work established immune-bone interactions, highlighting Treg-mediated bone protection. Mechanistically, Treg cells inhibit osteoclast differentiation via cytotoxic T-lymphocyte-associated protein 4/CD80/86 and IDO/tryptophan signaling while promoting osteoblast maturation (40, 41). Treg populations suppress osteoclastogenesis through Interleukin-4, Interleukin-10, and *transforming growth factor-beta (TGF-β)* secretion while enhancing bone formation (42). Furthermore, Treg cells stimulate *CD8+* T cell Wnt10b expression, activating osteoblast Wnt signaling to promote bone formation (43). Specific microbial taxa modulate Th17/Treg balance to influence bone turnover. *Clostridium clusters IV*, XIVa, and *XVIII* activate intestinal epithelial *TGF-β* production (44), while Bacteroides fragilis drives Th17 differentiation via Janus Kinase/Signal Transducer and Activator of Transcription 3 activation (45). Firmicutes populations suppress Treg differentiation while promoting Th17 expansion, upregulating stromal *RANKL* expression and expanding osteoclast precursor pools (46). B cells, as primary *RANKL* and *osteoprotegerin (OPG)* producers, respond to microbial regulation (47). Clostridium/*Bifidobacterium imbalances suppress* mechanistic target of rapamycin) signaling, elevating *RANKL/OPG* ratios. Bacteroides fragilis downregulates *OPG* via Wnt/β-catenin interference (48), promoting osteoclast differentiation. Bile acid metabolites demonstrate opposing effects: 3-oxoLCA inhibits Th17 differentiation through RAR-related Orphan Receptor Gammat binding, while isoalloLCA promotes Treg expansion via mitochondrial *reactive oxygen* sp*ecies (ROS)* induction (49), collectively orchestrating bone immune microenvironments.

#### 3.1.2 Inflammatory mediator regulation

Gram-negative *bacterial LPS* exerts context-dependent bone effects: promoting phagocyte differentiation from osteoclast precursors in *RANKL*-depleted conditions while enhancing osteoclastogenesis via Toll-like receptor activation in *RANKL*-sufficient environments (50). Compromised intestinal barrier integrity facilitates systemic *LPS* translocation, triggering immune activation and accelerated bone loss (51). *RANKL* functions as a critical immune-bone interface molecule expressed in activated T cells and mesenchymal lineages. Clostridium-derived secondary bile acids modulate inflammatory responses by suppressing *TNF-α*-induced immunity and inflammasome activation (52). Colitis models demonstrate elevated chemokine and cytokine levels (granulocyte colony-stimulating factor), *TNF-α*, Interleukin 12 p40 subunit, monocyte chemoattractant protein-1/C-C motif ligand 2, regulated on activation, normal T cell expressed and secreted/C-C Motif Chemokine Ligand 5, C-X-C motif chemokine ligand 1), stimulating osteoclast precursor proliferation and disrupting bone homeostasis (53).

### 3.2 Endocrine modulation

Gut microbiota regulate skeletal homeostasis through bioactive compound production, influencing endocrine networks governing bone metabolism (54, 55). Microbial communities orchestrate bone remodeling via estrogen-dependent and independent signaling cascades.

#### 3.2.1 Estrogen signaling

Estrogen receptor alpha mediates estrogen-dependent bone formation (56, 57), with estrogen receptor alpha deficiency reducing femoral length in female mice (58). Estrogen maintains bone mass through immune response regulation and osteoblast-osteoclast balance (59), mechanistically inhibiting *RANKL* expression in *CD3+* T cells and *CD20+* B cells while promoting osteoblast *OPG* production (60). Estrogen-deficient bone loss correlates with T cell-mediated *TNF-α* upregulation, indirectly enhancing osteoclastogenesis (61, 62). Gut microbiota critically influence estrogen-deficient osteoporosis. Ovariectomized rat models exhibit profound microbiota dysbiosis with elevated Firmicutes/*Bacteroidetes* ratios. microbiota profiling reveals positive correlations between *Ruminococcus, Clostridium*, and *Coprococcus* abundances with bone loss, contrasting with negative *Bacteroidetes* associations (63). Notably, germ-free and antibiotic-treated mice resist estrogen-deficient bone loss (64). Mechanistically, increased intestinal permeability drives dysbiosis and pathogen translocation, stimulating myeloid stromal cell secretion of *TGF-β, RANKL*, and *M-CSF* to promote osteoclast maturation.

#### 3.2.2 Serotonin-mediated pathways

Serotonin 5-hydroxytryptamine (5-HT) signaling critically regulates skeletal development and maintenance (65). Both osteoblasts and osteoclasts express 5-HT receptors, with elevated serotonin levels correlating with bone loss (66). Synthetic 5-HT inhibitors prevent ovariectomy-induced bone loss (67), with forkhead box O1 serving as a key mediator of intestinal 5-HT effects on osteoblast proliferation (68). Gut microbiota regulate bone metabolism via intestinal chromaffin cell 5-HT synthesis modulation (68). Luminal *SCFAs* promote tryptophan hydroxylase 1 mRNA expression and chromaffin cell 5-HT synthesis (69). Specifically, *Lactobacillus*, Streptococcus, and *Escherichia* coli metabolites induce tryptophan hydroxylase 1 expression in chromaffin cells, elevating peripheral 5-HT in germ-free mice (70). Chromaffin-derived 5-HT activates osteoblast precursor 5-HT1B receptors, promoting osteoclastogenesis while inhibiting osteoblast proliferation and bone formation (71).

#### 3.2.3 Parathyroid hormone signaling

Primary hyperparathyroidism, characterized by excessive PTH secretion (72), drives osteoporosis through osteocyte-derived *RANKL* and T cell-derived Interleukin-17A-mediated bone catabolism (73). Continuous PTH fails to induce bone loss in antibiotic-treated or germ-free mice. Segmented filamentous *bacteria* enable PTH-mediated expansion of intestinal *TNF+* T cells and Th17 populations with subsequent bone marrow recruitment. Bone marrow *TNF+* T cells upregulate C-C motif chemokine ligand 20 expression, facilitating intestinal-to-bone marrow Th17 cell migration (74). Intermittent PTH enhances bone formation through osteoblast Wnt pathway activation, promoting osteoblast formation, survival, and bone lining cell reactivation. Butyrate critically mediates intermittent PTH-induced skeletal anabolism (75), binding dendritic cell G protein-coupled receptor 43 to induce Treg differentiation. Subsequently, Treg cells promote bone marrow *CD8+* T cell Wnt10b expression, activating Wnt-dependent bone formation (75).

## 4 Protective mechanisms of gut microbiota and metabolites against osteoporotic progression

**Figure 3:**
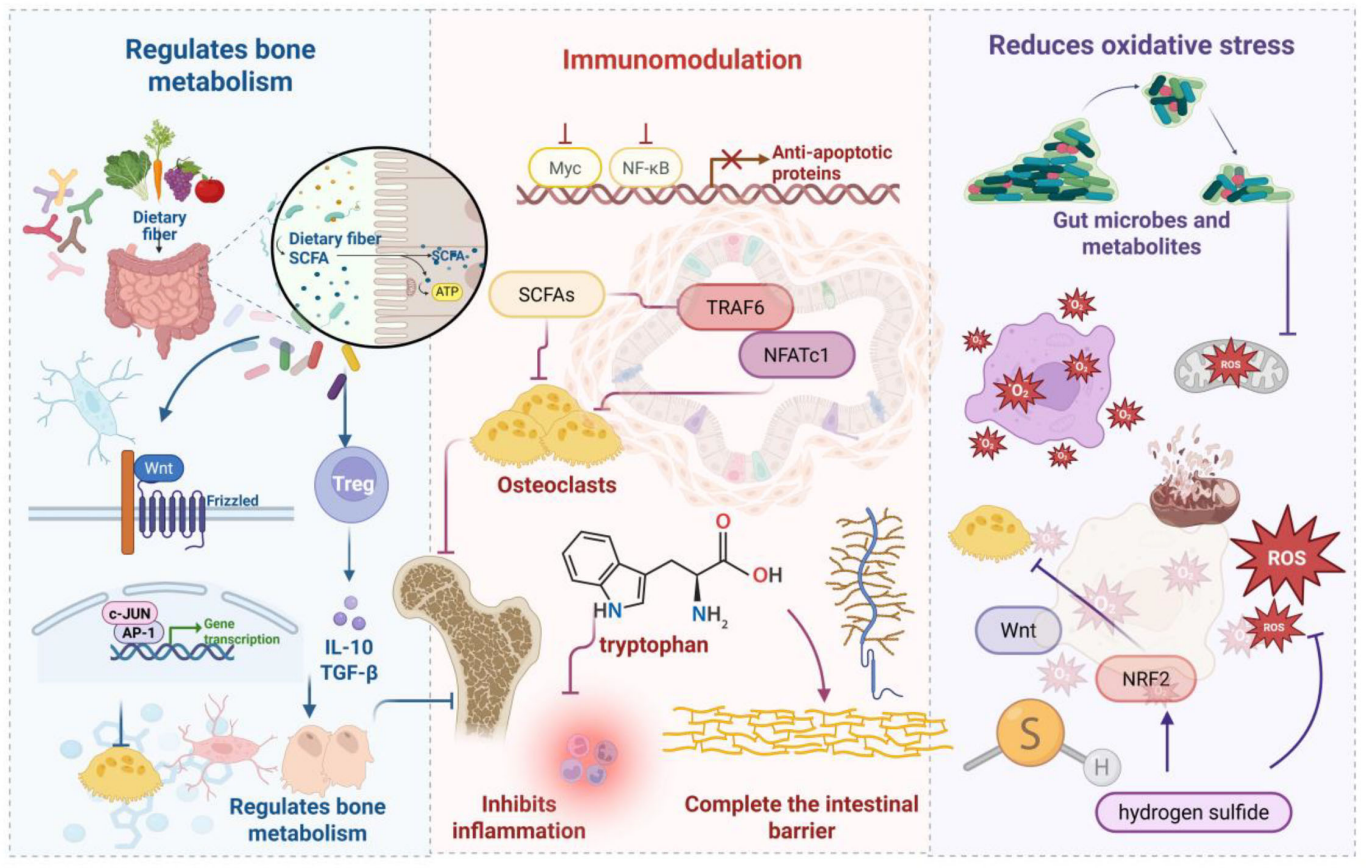
Gut microbiota-mediated osteoprotective mechanisms. *SCFAs* represent critical microbial metabolites that regulate bone homeostasis via Wnt signaling and regulatory T cell modulation. Additionally, tryptophan derivatives and *SCFAs* attenuate inflammatory responses while upregulating tight junction proteins to preserve intestinal barrier integrity, collectively inhibiting osteoporotic progression. microbiota-derived compounds reduce oxidative stress levels, providing anti-inflammatory and antioxidant benefits that suppress bone catabolism.

Osteoporotic pathogenesis involves complex regulatory imbalances across multiple molecular networks and signaling cascades. Emerging evidence demonstrates that targeted modulation of bone metabolism regulators, inflammatory mediators, and immune system components effectively attenuates osteoporotic progression. **Figure 3** illustrates the mechanistic framework whereby gut microbiota and their metabolites confer skeletal protection.

### 4.1 Bone metabolic regulation

*SCFAs* constitute pivotal microbial metabolites orchestrating bone homeostasis through multi-pathway mechanisms, principally via osteoclastogenesis inhibition and osteoblast function enhancement. *Butyrate* promotes stromal cell osteogenic differentiation and mineralization nodule formation (76, 77), primarily through Wnt signaling pathway activation—a central regulator of bone mass acquisition and maintenance (78). Gain-of-function Wnt mutations generate high bone mass phenotypes, whereas loss-of-function variants cause low bone mass and premature osteoporosis (79). *SCFAs* induce naïve *CD4+* T cell differentiation toward regulatory Tregs, with probiotics such as *Lactobacillus* rhamnosus GG conferring bone protection via the *SCFAs*-Tregs-bone metabolism axis. L. rhamnosus GG supplementation expands *SCFA*-producing *bacterial* populations, elevating intestinal and systemic *butyrate* concentrations to promote bone formation and increase trabecular volume (43). *Enhanced lactobacilli* and bifidobacteria levels facilitate mineral absorption, improving bone density (80). Gut microbiota composition modulates mineral bioavailability, particularly calcium, through intestinal pH regulation. Furthermore, gut microbiota participate in vitamin B and K biosynthesis alongside bile acid metabolism (81). Vitamins B and K represent essential bone health modulators (82), while bile acids critically regulate calcium absorption. The gut microbiota degrades macromolecular substrates into bioavailable components, supporting bone health and metabolic homeostasis while inhibiting osteoporotic progression (83).

### 4.2 Immunomodulation

Indole derivatives, as key tryptophan metabolic products, maintain gut microecological balance through multifaceted mechanisms. Molecularly, these compounds upregulate tight junction protein and mucoprotein expression in intestinal epithelial cells while enhancing anti-inflammatory Interleukin-10 production and suppressing pro-inflammatory Interleukin-8 expression, thereby modulating bone metabolism (84). Indole stimulates antimicrobial peptide and mucoprotein production, promotes intestinal villus cell proliferation, and inhibits pathogenic expansion to preserve mucosal barrier integrity (85). Concurrently, indole exerts anti-inflammatory effects through immune response modulation (86), with appropriate supplementation effectively attenuating inflammatory responses and optimizing microbiota composition (87). *SCFAs* represent another critical class of anti-inflammatory metabolites derived from indigestible dietary fiber fermentation. These compounds suppress autoimmune inflammation by inhibiting nuclear factor κB activation in B cells (88). Specifically, propionate and *butyrate* inhibit osteoclastogenesis and bone resorption through *TNF* receptor-associated factor 6 and nuclear factor of activated T-cells, cytoplasmic 1 downregulation, promoting bone density enhancement (14, 17). *Butyrate* significantly suppresses osteoclast activity via metabolic reprogramming in osteoclast precursors, enhancing glycolysis while downregulating key osteoclastic genes at the expense of oxidative phosphorylation (77). Additionally, *SCFAs* regulate osteoclast precursor survival, inducing programmed cell death to improve bone density without compromising osteoblast function (77), thereby critically modulating bone metabolic equilibrium.

### 4.3 Oxidative stress attenuation

Oxidative stress—characterized by imbalanced *ROS* generation and clearance—represents a pathological driver of cellular damage. Accumulating evidence establishes oxidative stress as a critical mediator of osteoporotic pathogenesis. Elevated *ROS* levels directly compromise bone tissue integrity through mitochondrial dysfunction, *DNA* fragmentation, and protein oxidative modifications, culminating in osteoblast apoptosis and diminished bone formation capacity. Simultaneously, *ROS* activate *nuclear factor kappa-light-chain-enhancer of activated B cells (NF-κB)* and mitogen-activated protein kinase signaling cascades, upregulating *RANKL* expression and enhancing osteoclast differentiation and activity. This process accelerates bone resorption, disrupting formation-resorption coupling and ultimately causing bone loss with trabecular architectural deterioration (89, 90). Within this pathological framework, antioxidant mechanisms function as negative regulatory circuits providing essential bone protection. Gut microbiota and their metabolites attenuate oxidative stress through multiple pathways, indirectly preventing osteoporotic progression. Probiotic strains including lactobacilli and bifidobacteria upregulate key antioxidant enzymes—superoxide dismutase, catalase, and glutathione peroxidase—enhancing host free radical scavenging capacity. These strains additionally produce small-molecule antioxidants such as glutathione and extracellular polysaccharides, further augmenting antioxidant defenses (91). Gut-derived metabolites demonstrate substantial antioxidant regulatory potential. *Butyrate* activates nuclear factor erythroid 2–related factor 2/heme oxygenase-1 pathways to promote antioxidant factor synthesis, significantly reducing *ROS* levels while protecting bone cells from oxidative damage. Propionate suppresses endogenous *ROS* generation via nicotinamide adenine dinucleotide Phosphate oxidase inhibition while upregulating mitochondrial antioxidant enzyme expression, preserving mitochondrial homeostasis and bone cell functionality. Hydrogen sulfide, another key metabolite, promotes bone marrow mesenchymal stem cell proliferation and osteogenic differentiation through Wnt/β-catenin pathway activation, while demonstrating bone-protective effects via *RANKL* expression suppression and osteoclast formation inhibition (92).

### 4.4 Contextual and dose-dependent effects of gut metabolites

An increasing body of evidence indicates that certain canonical gut-derived metabolites do not consistently exert osteoanabolic or anti-resorptive effects on bone metabolism. Instead, they exhibit marked dose dependence and sensitivity to host-specific physiological contexts. Their biological effects are regulated in a dynamic manner by multiple factors, including metabolite concentration, the inflammatory microenvironment, endocrine signaling, and immune equilibrium. These context-dependent phenomena are especially pronounced in SCFAs, such as butyrate, and endotoxins like LPS (93). Butyrate, a SCFA possessing significant immunomodulatory and tissue-regenerative properties, demonstrates a characteristic biphasic influence on bone metabolism. At low concentrations (<1 mM), it inhibits histone deacetylase activity, activates osteogenic Wnt signaling pathways, promotes Wnt10b expression, and induces osteogenic differentiation in bone marrow mesenchymal stem cells, thus enhancing osteogenesis (93). Additionally, butyrate contributes indirectly to skeletal homeostasis by expanding Tregs and suppressing Th17-mediated inflammation (94). However, at higher concentrations (>5 mM), butyrate facilitates the release of proinflammatory cytokines such as TNF-α and IL-6 from monocyte–macrophage lineages, leading to RANKL-mediated osteoclastogenesis (93). Under inflammatory conditions, it may further amplify osteoclast precursor activation and bone resorption via macrophage pyroptosis (93). This dose-dependent shift in bioactivity underscores the need for precise dosage control and personalized contextual evaluation when considering butyrate as a therapeutic agent. Similarly, LPS—a major constituent of the Gram-negative bacterial outer membrane—can translocate into the systemic circulation in the event of impaired gut barrier integrity and significantly reshape the bone immune microenvironment. At low doses, in the absence of RANKL co-stimulation, LPS primarily drives precursor cells toward phagocytic differentiation with limited osteoclastogenic capacity, potentially contributing to immune homeostasis. In contrast, elevated LPS concentrations activate the TLR4/MyD88/NF-κB signaling cascade, leading to overproduction of RANKL and proinflammatory cytokines and thereby accelerating osteoclastogenesis and bone resorption. Importantly, these dose–response effects are not universally consistent across physiological states. They are strongly influenced by host immune profiles, hormonal status, and gut microbiota composition. For example, under estrogen-deficient conditions, increased gut permeability and Treg/Th17 imbalance may amplify the bone-resorptive potential of inflammatory metabolites. Concurrently, changes in gut pH can alter the ionization state and membrane transport efficiency of SCFAs, thereby modifying their bioactivity within the bone microenvironment (94). Collectively, the modulatory roles of gut metabolites in bone metabolism can be conceptualized as a tri-axial “dose–effect–context” interaction model. This framework not only elucidates the bidirectional nature of their biological actions but also provides a theoretical foundation for the development of dose-optimized, host-tailored microbiota-targeted therapeutic strategies.

## 5 Translational applications of gut microbiota in osteoporosis management

**Figure 4:**
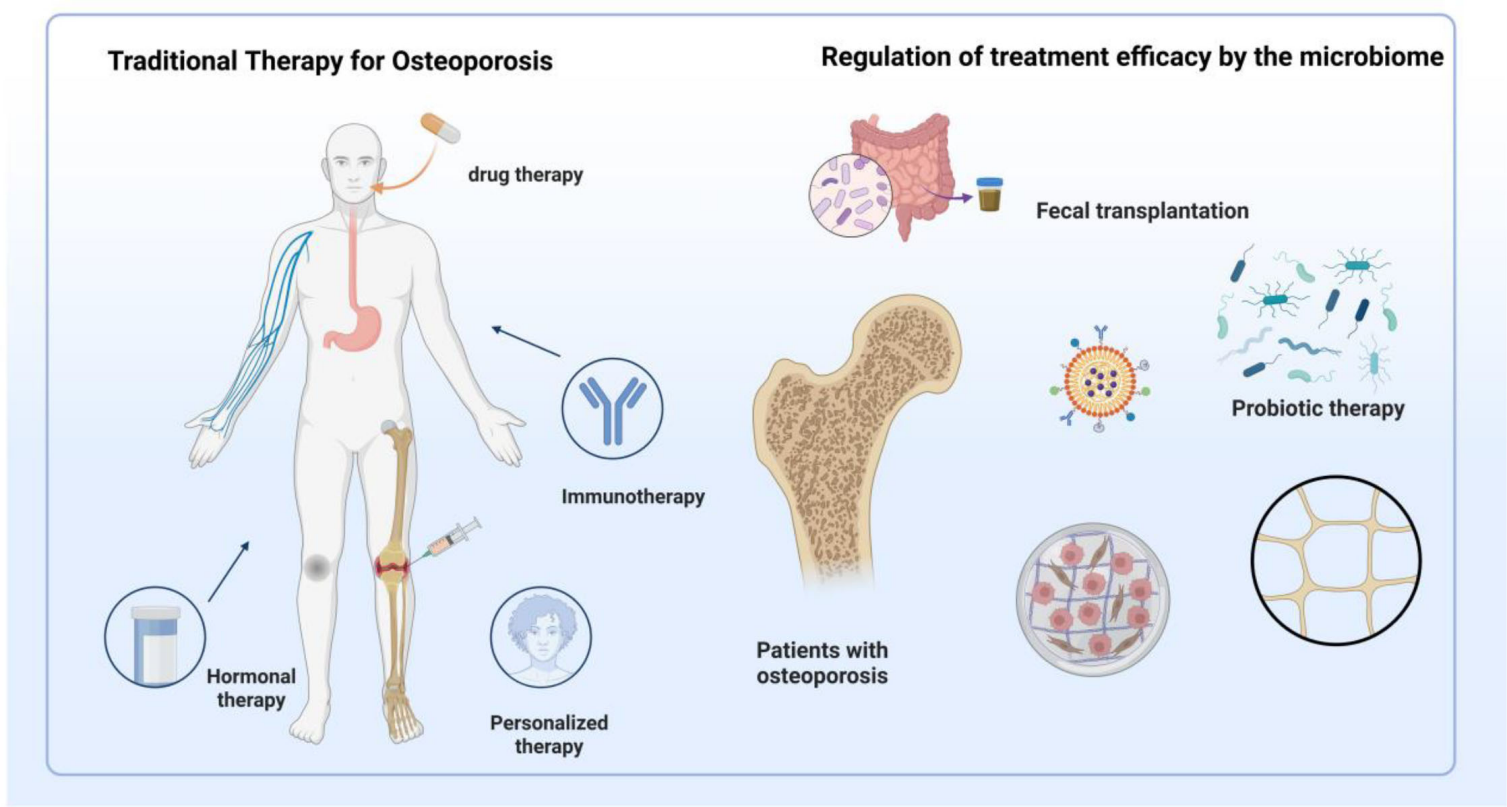
Traditional therapies and microbiota-based interventions for osteoporosis treatment. This figure illustrates two primary approaches to osteoporosis management. On the left, traditional therapies include drug therapy (e.g., bisphosphonates, hormone replacement), immunotherapy, hormonal regulation, and personalized medical strategies targeting bone turnover. On the right, microbiota-targeted interventions are shown to influence therapeutic efficacy through mechanisms such as *fecal microbiota transplantation* (*FMT*), probiotic supplementation, and microbial metabolite regulation. These strategies aim to restore gut microecological balance, enhance intestinal barrier integrity, modulate immune responses, and regulate endocrine factors, thereby contributing to improved skeletal health in osteoporotic patients.

Contemporary osteoporosis therapeutics encompass bone resorption inhibitors and formation promoters, including bisphosphonates, calcitonin, and estrogen replacement therapy. However, adverse effects and prohibitive treatment costs limit clinical implementation. Recent advances in gut microbiota-bone health research have established microbiota modulation as a promising therapeutic target for osteoporotic intervention. **Figure 4** illustrates gut microbiota-based versus conventional therapeutic approaches.

### 5.1 Microbiota-based osteoporosis prevention

Probiotic interventions prevent osteoporotic progression through gut microbiota homeostasis restoration, facilitating vitamin and mineral absorption, immune system enhancement, and bioactive metabolite production, including organic acids and amino acids (74). *Clostridium butyricum CBM588* metabolizes dietary fiber to generate protective *SCFAs*, attenuating bone loss. Faecalibacterium prausnitzii prevents inflammation-mediated bone catabolism via *NF-κB* inhibition and Interleukin-8 suppression while upregulating protective Interleukin-10 and Interleukin-12 expression (74). Preclinical studies demonstrate that lactobacilli and bifidobacteria effectively prevent ovariectomy-induced bone loss through tartrate-resistant acid phosphatase positive, receptor activator of nuclear factor κB positive, and *RANKL+* cell formation inhibition. Mechanistically, propionate and *butyrate* reduce osteoclast populations and serum C-terminal telopeptide of type I collagen levels, while acetate suppresses osteoclastogenesis via T cell and B cell functional modulation (74).

Microbiota-mediated nutrient absorption regulation represents a fundamental mechanism influencing bone remodeling (95). Gut microbiota orchestrate food digestion, energy recovery, and vitamin biosynthesis and absorption. Comparative germ-free versus conventional mouse studies reveal microbiota-enhanced intestinal monosaccharide absorption and improved dietary energy extraction efficiency (96). Additionally, gut communities synthesize essential vitamins K and B complex, maintaining physiological vitamin requirements. Microbiota further regulate calcium bioavailability through intestinal pH modulation and calcium solubility enhancement (97), promoting skeletal health.

### 5.2 Microbiota-based osteoporosis therapeutics

Traditional osteoporotic management strategies focus on bone density improvement and fracture risk reduction through anti-resorptive and anabolic agents, yet demonstrate inherent limitations. Emerging gut homeostasis-targeting approaches offer unique therapeutic advantages, providing innovative osteoporotic intervention paradigms.

#### 5.2.1 Microbiota-mediated therapeutic mechanisms

Gut microbial metabolites demonstrate substantial osteoprotective potential. Tryptophan derivatives, including indole-3-acetic acid and indole propionic acid, modulate intestinal barrier integrity via aryl hydrocarbon receptor activation, influencing bone catabolism. *SCFAs* critically regulate osteoporotic progression (C. 98), with acetate restoring aged bone marrow mesenchymal stem cell osteogenic capacity through cytoplasmic acetyl-coenzyme A restoration and chromatin accessibility enhancement. *Akkermansia* and *Eubacterium* species generate propionate, while *Clostridiaceae* and *Eubacteriaceae* produce *butyrate*, promoting bone formation via Treg-dependent mechanisms (98). Plant fiber-enriched dietary supplementation elevates *SCFA* levels, supporting skeletal homeostasis maintenance.

#### 5.2.2 Microbiota-targeting therapeutic strategies

Probiotic interventions exert therapeutic effects through three principal mechanisms: intestinal epithelial barrier strengthening via tight junction protein regulation and permeability reduction (99); immunomodulation through Treg/Th17 balance restoration, exemplified by Bacillus clausii-mediated bone marrow and splenic Treg expansion alongside Th17 suppression, and *Lactobacillus* rhamnosus *GG/L*. reuteri-mediated Treg functional enhancement (100); and sex hormone regulation through sterol microbiota modulation, whereby microbial β-glucuronidase and β-glucosidase encoding influences estrogen metabolism and local/systemic hormone levels (100). Clinical applications demonstrate therapeutic efficacy: Bacteroides vulgatus *ATCC* 8482 reduces *TNF-α* via *LPS/TLR4/NF-κB* pathway downregulation (101), while *Lactobacillus plantarum NK3*/*Bifidobacterium longum NK49* combinations suppress NFκB/*TNF-α* signaling (102). Clinical trials confirm that probiotic supplementation reduces bone resorption markers and enhances bone density in postmenopausal women (103). Fracture healing applications show promise: *Akkermansia muciniphila facilitates* healing through intestinal permeability reduction and inflammatory attenuation (104), while bifidobacteria promote barrier function, alleviate fracture-associated inflammation, and accelerate callus cartilage remodeling. Clinical microbiota therapy demonstrates significant potential. Prebiotic fibers, including inulin and fructooligosaccharides, demonstrate bone health benefits through beneficial *bacterial* proliferation promotion, intestinal barrier enhancement, and immune response modulation, indirectly supporting bone metabolism. These compounds increase bone density and reduce resorption, particularly benefiting elderly and osteoporotic populations (105, 106). Synbiotic formulations combining probiotic-prebiotic advantages represent emerging osteoporotic interventions, demonstrating superior efficacy compared to individual components through synergistic gut health improvement and beneficial *bacterial* growth promotion. However, clinical translation challenges persist in microbiota osteoporotic therapy. Despite preclinical validation in animal models, clinical application verification remains insufficient. Existing trials predominantly feature small sample sizes with inadequate statistical power, compromising conclusion reliability and generalizability. Future investigations require large-scale, randomized controlled, long-term clinical trials to ensure microbiota intervention efficacy and safety.

#### 5.2.3 Gut microecology reconstruction

*FMT* represents a novel therapeutic modality that reconstructs gut microecological balance through healthy donor microbiota transfer to patients (107). Successful applications provide innovative osteoporotic microbial treatment paradigms, with *FMT* influencing bone metabolism through microbiota compositional regulation, intestinal barrier improvement, and immune system modulation (108). Nevertheless, *FMT* osteoporotic applications face substantial challenges. Primary safety concerns include segmented filamentous bacteria introduction potentially inducing Th17 development via Interleukin-17/Interleukin-23 pathways, exacerbating bone loss (109). Donor microbiota heterogeneity may generate therapeutic variability, necessitating standardized screening criteria and transplantation protocol optimization. *FMT*-probiotic combination strategies require investigation for enhanced therapeutic outcomes through optimized microbiota composition, though clinical data remain limited. *FMT* clinical implementation involves safety risks, including infectious disease transmission, adverse immune reactions, and long-term dysbiosis-related complications. While rigorous donor screening, pathogen detection, and standardized preparation protocols mitigate risks, protocol variability across institutions maintains safety concerns. In 2023, the U.S. Food and Drug Administration issued a safety alert highlighting incidents of severe infections, including sepsis, in recipients of FMT due to multidrug-resistant organisms, emphasizing the critical need for stringent screening and quality control of donor microbiota in clinical settings (110). Elderly individuals, a high-risk group for osteoporosis, exhibit age-related declines in immune function, increasing their susceptibility to FMT-associated adverse events (111). Therefore, it is recommended that future clinical trials evaluating the efficacy of FMT extend follow-up durations beyond 12 months to monitor potential long-term risks 112). Further research may focus on constructing a quantitative framework to assess risk–benefit profiles based on host microbiota composition, immune function, and donor–recipient compatibility, thereby enabling personalized safety evaluations in bone metabolic disorders. Notably, variations in donor screening and preparation protocols across institutions pose persistent challenges to the standardization of FMT. Future investigations should validate its safety under more stringent clinical conditions and develop harmonized and standardized procedural guidelines.

#### 5.2.4 Clinical translation

microbiota-based interventions have emerged as a promising strategy for the clinical management of osteoporosis. In a randomized, double-blind, placebo-controlled clinical trial, Nilsson et al. ‘s randomized, double-blind, placebo-controlled trial revealed *Lactobacillus reuteri-mediated* bone density enhancement in elderly populations (113). Lambert et al. demonstrated that probiotic-red clover extract combinations (rich in isoflavone aglycones) significantly attenuate estrogen deficiency-induced bone loss through beneficial estrogen metabolite promotion. Synergistic effects with calcium, magnesium, and calcitonin supplementation exceed monotherapy efficacy (114), establishing theoretical foundations for probiotic-prebiotic-mineral matrix combinations. Ovariectomy-induced osteoporosis models reveal reduced aryl hydrocarbon receptor ligand levels, prompting indole acetic acid and indole propionic acid supplementation strategies. Integrated 16S rRNA sequencing, targeted *HPLC-QQQ-MS* metabolomics, and biological assessments elucidate tryptophan metabolite-mediated gut-bone axis improvements, providing novel osteoporotic therapeutic development frameworks (98). **Table 1** is summary of clinical and translational studies on microbiota-based Interventions for bone health. Despite these encouraging findings, existing clinical trials are constrained by limited sample sizes, brief follow-up periods, and considerable heterogeneity in probiotic strains and dosing regimens. Consequently, the overall quality of evidence remains low to moderate. There is an urgent need for large-scale, multicenter, long-term randomized controlled trials to rigorously assess the effects of microbiota-targeted therapies on critical clinical outcomes, including bone mineral density and fracture incidence. Furthermore, the incorporation of the grading of recommendations assessment, development and evaluation framework is essential to enhance the scientific rigor and translational credibility of evidence supporting microbiota-based approaches for osteoporosis treatment.

**Table 1:** Summary of clinical and translational studies on microbiota-based interventions for bone health.

Study	Intervention	Population and sample size	Follow-up duration & study design	Primary outcomes	Grade evidence level
(113)	Lactobacillus reuteri ATCC PTA 6475, 1×10^10^ CFU/day, oral administration	Women aged ≥70 years, n = 90	12 months, double-blind RCT	Significantly reduced trabecular bone loss and improved femoral bone mineral density	Moderate
(114)	Probiotic + isoflavone complex (including red clover extract)	Postmenopausal women with bone loss, n = 85	12 weeks, double-blind RCT	Improved estrogen metabolism and decreased bone resorption markers	Low
(98)	Association analysis of tryptophan metabolites (indole-3-acetic acid, indolepropionic acid) with bone status	Chinese pre- and postmenopausal women, n = 54 (32 pre/22 post)	Single sampling, observational cohort study	Marked reduction in metabolites in postmenopausal women, suggesting potential targets for intervention	Very Low

## 6 Summary and prospects

Gut microbiota and their metabolites significantly influence osteoporotic pathogenesis, establishing causal relationships between microbial dysbiosis and disease development. Pathogenic bacteria and associated metabolites demonstrate elevated levels in osteoporotic patients, promoting disease progression through inflammatory induction, osteoclastogenesis enhancement, and endocrine disruption. Conversely, *Lactobacillaceae strains*, bifidobacteria, and *SCFAs* show reduced abundance in osteoporotic individuals yet exhibit protective effects through inflammation suppression, osteoclast inhibition, and bone remodeling optimization.

Gut microbiota and metabolites demonstrate therapeutic potential in osteoporosis management, with probiotics and postbiotics showing promising applications in disease prevention and treatment. Specific microorganisms with anti-osteoporotic properties represent potential pharmacological targets for therapeutic intervention. Despite preliminary mechanistic insights into gut microbiota-osteoporosis interactions, specific regulatory mechanisms require further elucidation. Current research predominantly examines individual bacterial species or metabolites, while gut microbiota constitute complex ecosystems featuring intricate inter-microbial and host-microbe interaction networks. Future investigations must comprehensively characterize bacterial species interactions and host immune-metabolic pathway integration to understand microbiota-mediated osteoporotic regulation. Critical knowledge gaps persist regarding microbiota metabolite cellular recognition and downstream signaling cascade regulation. While existing research reveals preliminary bone metabolic effects, specific cellular mechanisms and signaling pathways—particularly bone resorption-formation balance regulation—require clarification. Future studies should precisely elucidate metabolite-mediated bone metabolism regulatory mechanisms across diverse physiological contexts.

Although microbiota modulation demonstrates osteoporotic improvement potential in animal studies, clinical mechanisms and efficacy require validation. Future research should establish comprehensive animal models integrating transcriptomics, proteomics, and multi-omics technologies for systematic microbiota-metabolite mechanism investigation. This comprehensive approach will reveal condition-specific microbiota functional and metabolic changes affecting osteoporosis, providing precise theoretical foundations for clinical interventions. Multi-level investigations encompassing small molecule screening, animal validation, and clinical trials should evaluate gut microbiota-based therapeutic strategies. Exploring microbiota-drug metabolism interactions will provide individualized treatment insights. Through comprehensive microbiota regulatory strategy exploration, future research may establish novel osteoporotic therapeutic targets and interventions, advancing clinical field applications.
